# Peutz-Jeghers syndrome in gynecological cancers: bibliometric trends, clinical insights, and future directions

**DOI:** 10.1016/j.gore.2025.101941

**Published:** 2025-09-04

**Authors:** Tianhui Niu, Jinghui Jia, Lei Zhang, Hongfang Wang, Guoqing Cai

**Affiliations:** Department of Obstetrics, Gynecology and Reproductive Sciences, Air Force Medical University, Air Force Medical Center, PLA, Beijing, China

**Keywords:** Peutz-Jeghers syndrome, Gynecological malignancies, Bibliometric analysis, STK11 gene, Gastric-type endocervical adenocarcinoma, Molecular pathogenesis

## Abstract

**Background:**

Peutz-Jeghers syndrome (PJS) is an autosomal dominant disorder caused by germline mutations in the STK11 tumor suppressor gene. It is characterized by mucocutaneous pigmentation, gastrointestinal polyposis, and a significantly elevated risk of various malignancies. While the association between PJS and gastrointestinal cancers is well established, its relationship with gynecological malignancies, particularly gastric-type endocervical adenocarcinoma (G-EAC), remains poorly understood. The rarity and clinical heterogeneity of PJS have hindered the development of evidence-based guidelines, especially for gynecological tumors.

**Methods:**

We conducted a comprehensive bibliometric analysis of 96 English-language publications from 2010 to 2024 to map global research trends on PJS-related gynecological cancers, identifying key topics and tracing the evolution of knowledge in this field. This was complemented by a retrospective case series analysis of PJS patients diagnosed with G-EAC, integrating clinical, imaging, and pathological data to characterize their disease features.

**Results:**

The bibliometric analysis revealed a growing research focus on STK11 molecular pathogenesis and improved surveillance strategies for PJS. Our case series of 10 PJS patients with G-EAC underscores the early onset and aggressive nature of this malignancy—all cases were HPV-negative. Clinical presentation often included watery vaginal discharge and pelvic pain, with imaging showing cervical masses and pelvic effusions. Pathological review confirmed G-EAC in six patients and atypical lobular endocervical glandular hyperplasia (aLEGH) in others, highlighting the need for heightened vigilance in monitoring PJS patients.

**Conclusion:**

These findings emphasize the urgent need for individualized, multidisciplinary screening and management strategies for PJS-associated gynecological cancers. Improved awareness of PJS-related G-EAC is crucial for early detection. Integration of genetic counseling, advanced imaging, and molecular diagnostics into routine care for PJS patients will help optimize outcomes in this high-risk population.

## Highlights

• PJS significantly increases gynecological cancer risk, especially G-EAC.
• Research focus shifts from genetics to molecular pathogenesis and clinical strategies.
• Enhanced screening and multidisciplinary care improve early detection and outcomes.

## 1 Introduction

Peutz-Jeghers syndrome (PJS) is an autosomal dominant disorder caused by germline mutations in the tumor suppressor gene STK11. Clinically, it is characterized by mucocutaneous pigmentation, gastrointestinal polyposis, and a significantly elevated risk of various malignancies (Sato et al., 2022, Latchford and Clark, 2022). While the association between PJS and gastrointestinal cancers is well established, its relationship with gynecological malignancies, particularly gastric-type endocervical adenocarcinoma (G-EAC) remains poorly understood (Lu et al., 2021, Nishio et al., 2024). G-EAC is a rare and non-human papillomavirus (HPV)-associated mucinous adenocarcinoma of the uterine cervix that has emerged as a particularly aggressive and under-recognized entity in the context of PJS (Nishio, 2023). It constitutes only 10–15 % of all cervical adenocarcinomas in the general population,but its incidence is markedly higher among PJS patients. Epidemiologic studies estimate that 15–30 % of women with PJS will develop G-EAC during their lifetime, and conversely, approximately 10 % of all G-EAC cases are linked to PJS (Stolnicu et al., 2018, Park et al., 2021). This underscores PJS as a major risk factor for G-EAC. Importantly; G-EAC in PJS often presents at a younger age than sporadic cervical adenocarcinomas, frequently occuring during a patient’s reproductive years, and tends to be more advanced and chemoresistant at diagnosis. These features highlight the need for specialized screening and management protocols tailored to women affected by PJS.

Despite the clinical significance of PJS-related gynecological cancers, the literature remains limited due to the rarity of PJS, the heterogeneity of clinical manifestations, and the absence of large prospective studies. These factors have hindered the development of evidence-based clinical guidelines, especially for gynecological tumors (Gordhandas et al., 2022, Lin et al., 2025). To address this knowledge gap, we conducted a comprehensive bibliometric analysis aimed at mapping global research trends, identifying key research themes, and tracing the evolution of knowledge in the field of PJS-related gynecological cancers (Wu et al., 2022). This was supplemented by a detailed case series analysis of patients diagnosed with PJS-associated G-EAC, offering clinical validation and highlighting unique pathological and prognostic features. By integrating both macro-level literature insights and micro-level clinical data, this study provides a novel perspective on the pathogenesis, clinical trajectory, and management complexities of PJS-associated gynecological malignancies.

## 2 Methods

### 2.1 Literature search strategy

**Fig. 1:**
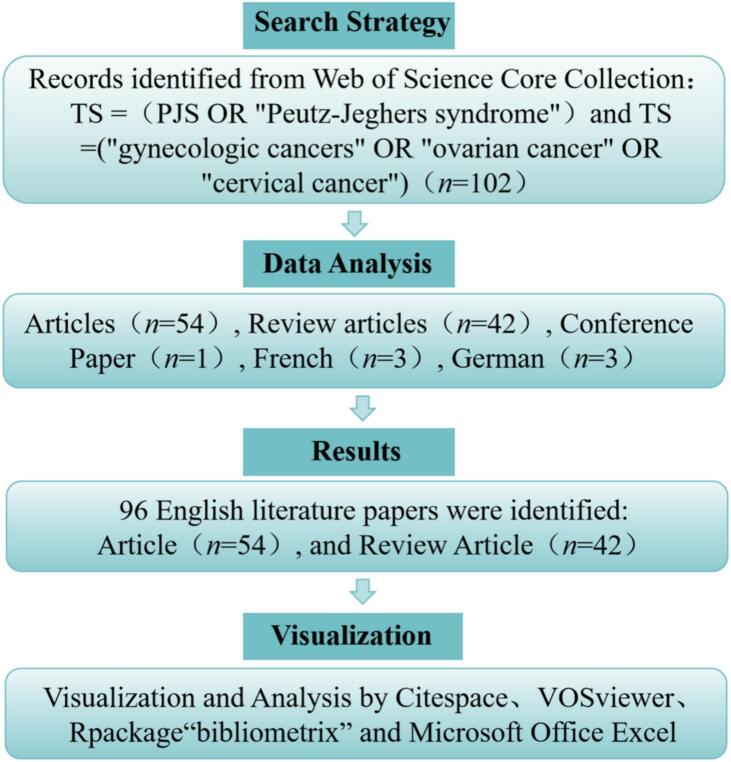
Flowchart of the literature selection process. A schematic representation of the step-by-step procedure used to identify and select relevant publications on PJS and gynecological cancers from the initial database search to the final inclusion of articles for analysis.

We conducted a systematic literature search using the Web of Science Core Collection (WoSCC) database to identify studies on PJS and gynecological malignancies. The search query combined terms for PJS (including “PJS” and “Peutz-Jeghers syndrome”) with terms for gynecologic cancers (“gynecologic cancers”, “ovarian cancer”, “cervical cancer”), as detailed in the original report. Only English- language studies published between January 1, 2010, and December 31, 2024 were included. Document types were restricted to peer-reviewed articles and review papers. The retrieval was performed in a single day (February 23, 2025) to avoid bias from database updates. After removing duplicates, a total of 96 records were included for analysis. Fig. 1 presents a detailed screening flowchart used in this study.

### 2.2 Bibliometric analysis

We employed bibliometric software tools to analyze the publication data. CiteSpace (version 6.3.R1) was utilized with a one-year time slicing interval to detect temporal trends and identify keywords with strong citation bursts, indicating emerging research frontiers and paradigm shifts over time (Synnestvedt et al., 2005). In the generated visualizations, nodes represent entities such as countries, institutions, journals, or authors,node size reflects frequency, while color denotes classification. The thickness of connecting lines indicates the strength of collaboration or co-citation relationships among these items (Ninkov et al., 2022). VOSviewer (version 1.6.20) was used to construct and visualize large-scale networks, mapping collaborations between countries and institutions, as well as keyword co-occurrence patterns to identify major research clusters (van Eck and Waltman, 2010). Additionally, the R package “bibliometrix” was used to perform thematic evolution analysis, illustrating how research themes have shifted from 2010 to 2024.

### 2.3 Case series study

We retrospectively reviewed the medical records of female PJS patients treated at a single institution (Air Force Medical Center) between October 2024 and June 2025. From a cohort of 60 female PJS patients, we identified those who developed cervical glandular lesions or G-EAC. Inclusion criteria were a confirmed diagnosis of PJS and subsequent diagnosis of aLEGH (a precursor lesion) or G-EAC. Among the 60 PJS patients, we identified 10 who met the inclusion criteria (aLEGH or G-EAC). All 10 eligible patients were included in this series; no cases were excluded. Clinical data extracted included patient demographics, family history of PJS or cancer, age at PJS diagnosis and at cancer diagnosis, presenting symptoms, results of gynecologic examinations and imaging (ultrasound, MRI/CT), laboratory tests (HPV status, cytology), and pathological findings. This study was conducted in accordance with the Declaration of Helsinki and approved by the Institutional Ethics Committee of the Air Force Medical Center (Approval No. KT (Research) 2024–31-PJ01). Informed consent for inclusion was obtained from all patients or their legal guardians.

## 3 Results

### 3.1 Bibliometric analysis findings

#### 3.1.1 Publication trends

**Fig. 2:**
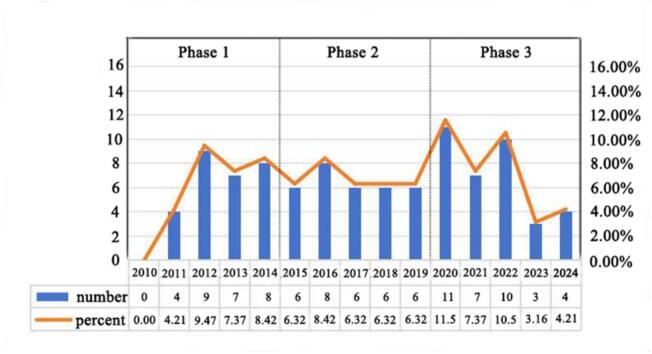
Annual trends in publications related to PJS and gynecological cancers from 2010 to 2024. A chronological visualization illustrating the yearly distribution of scholarly outputs in the field of PJS and gynecological cancers over the past 15 years.

Our search yielded 96 publications (54 original articles and 42 reviews) on PJS and gynecological tumors from 2010 to 2024. The annual publication output showed a three-stage pattern (Fig. 2). From 2010 to 2024, research output grew rapidly, rising from 0 to 9 publications per year, likely reflecting the establishment of foundational research frameworks in this area. A plateau phase followed from 2015 to 2019, with 6 to 8 publications annually, suggesting a period of consolidation or potential research bottlenecks. The most recent period (2020–2024) saw fluctuating output, peaking at 11 in 2020 and declining to 3 in 2023, possibly influenced by shifting research focus or external factors, such as the global pandemic. Despite recent fluctuations, the average of 7 publications per year over the last five years indicates sustained interest in the field.

#### 3.1.2 Geographic and institutional analysis

**Fig. 3:**
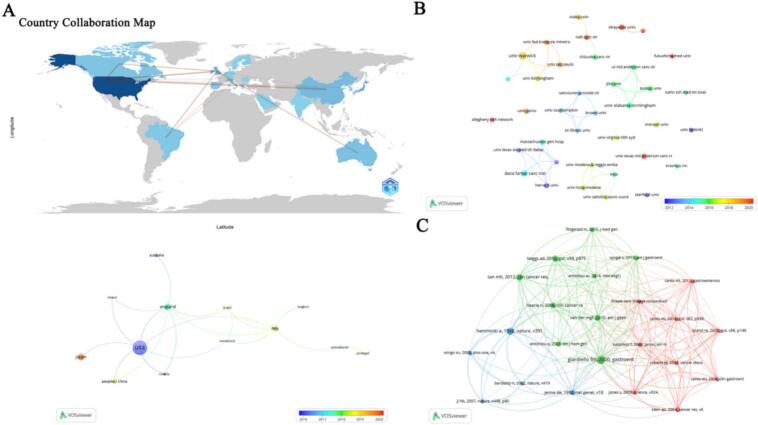
Geographic and institutional distribution and collaboration network in PJS and gynecological cancer research. A: Geographical distribution and international collaboration network among countries contributing to PJS and gynecological cancer research; B: Visualization of institutions involved in the study of PJS and gynecological cancers; C: Visualization of co-cited references in the literature on PJS and gynecological cancers.

Publications originated from 22 countries and 193 institutions. The United States led in output with 43 publications, followed by Japan (13) and the United Kingdom (12), collectively accounting for 60 % of the total (Table S1). A country level collaboration network (Fig. 3A) revealed active international partnerships, with the U.S. collaborating extensively with Japan, the UK, China, and others, and the UK also linking with China, Italy, and Australia. Japan’s collaborations were primarily with the U.S. At the institutional level, the top contributors were mostly in the UK and U.S (Table S1). The University of Warwick (UK) led with nine publications, followed by the Dana-Farber Cancer Institute (US) and University of Alabama at Birmingham (US). A network of institutions with ≥ 2 publications (Fig. 3B) showed strong domestic collaborations in the U.S., while the University of Warwick and Jilin University engaged in international collaborations. Notably, some high-output institutions, such as Okayama University in Japan, made significant contributions without recorded international co-authorships.

Over the past decade, 5700 citations were recorded for research on PJS and gynecological cancers. The top ten co-cited references received at least 13 citations (Table S2) (Giardiello et al., 2000, Hemminki et al., 1998, Tan et al., 2012, Beggs et al., 2010, van Lier et al., 2010, Hearle et al., 2006, Jenne et al., 1998, Antoniou et al., 2003, Syngal et al., 2015, Klein et al., 2004). Highly cited foundational work, such as the 2000 Gastroenterology study by Giardiello et al. (Giardiello et al., 2000), which highlighted the significantly elevated cancer risk in familial PJS, and the 2012 Clinical Cancer Research paper by Tan et al. (Tan et al., 2012), which quantified the lifetime cancer risks associated with germline PTEN mutations, has laid the groundwork for understanding the hereditary cancer predispositions in PJS (Fig. 3C). These influential studies likely contributed to the growing international interest in PJS-related gynecological malignancies and helped establish the U.S. as a leading contributor to this field of research.

#### 3.1.3 Journal collaboration networks

**Fig. 4:**
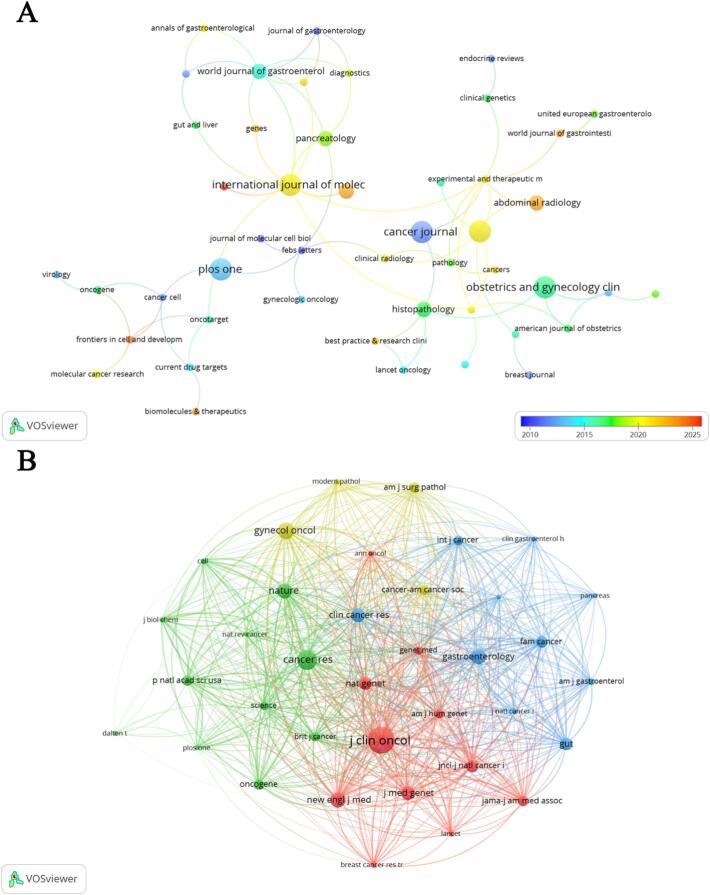
Journal network analysis in the study of PJS and gynecological cancers. A: Network map of journals publishing research on PJS and gynecological cancers; B: Clustered network map of co-cited journals.

Research on PJS and gynecological cancers was published across 76 journals, indicating a broad dissemination of research in this niche field. The most productive journals included International Journal of Molecular Sciences, Cancer Journal and Obstetrics and Gynecology Clinics of North America (Table S3). A journal collaboration network (Fig. 4A) showed that many top journals in this field are interconnected through shared citations. Table S3 listed the top 10 co-cited journals, with Journal of Clinical Oncology and Cancer Research emerging as the most frequently co-cited journals. The co-citation network (Fig. 4B) showed strong connections between Journal of Clinical Oncology and journals such as Cancer Research, Gynecologic Oncology, and Nature.

#### 3.1.4 Research hotspots and emerging themes

**Fig. 5:**
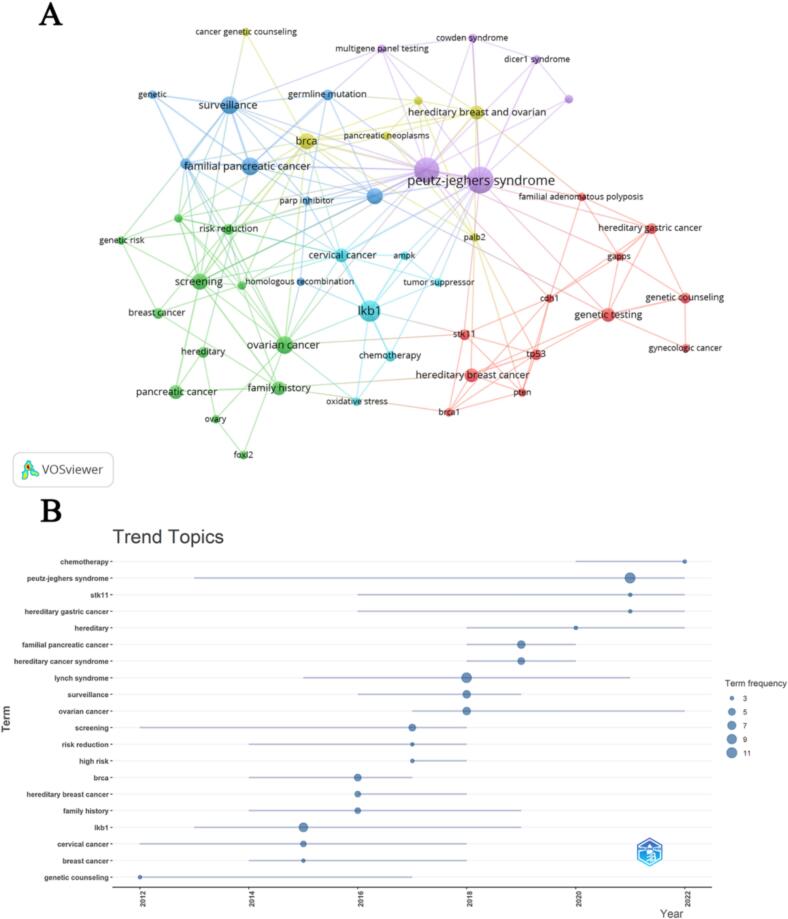
Co-occurrence analysis of keywords and topic evolution in global research on PJS and gynecological cancers. A: Cluster visualization of co-occurring keywords; B: Temporal evolution of research topics in the field of PJS and gynecological cancers.

Keyword co-occurrence and cluster analysis (Table S4) have identified several major research themes in PJS-related gynecological cancer research. A cluster map (Fig. 5A) grouped keywords into six distinct clusters, each corresponding to different focus areas. Early research centered on genetic counseling and familial cancer risk, reflecting an initial emphasis on identifying at-risk individuals and understanding inheritance. A landmark systematic review conducted by Van Lier et al. marked a pivotal moment in understanding the extensive cancer risks associated with PJS, revealing both the intricate genetic architecture involved and its profound implications for patient management (van Lier et al., 2010). By 2015, the research landscape underwent a significant transformation, shifting towards pathogenesis and exploring PJS within the context of other hereditary cancer syndromes. Epidemiological investigations have played a crucial role in delineating the cancer risk profile for individuals with PJS. Research conducted by Chen et al. provided critical quantitative insights, demonstrating that patients with PJS face substantially elevated cancer risks across multiple organ systems (Chen et al., 2017). In recent years, keywords like LKB1/STK11, surveillance, and screening have dominated, indicating a strong current emphasis on the molecular basis of PJS and strategies to detect cancers early. Researchers have made substantial advancements in characterizing STK11 gene mutations, elucidating the molecular mechanisms underlying cancer predisposition while also identifying complex genotype-phenotype correlations. Emerging frontiers include hereditary cancer and chemotherapy, suggesting new directions in both basic science and clinical management (Fig. 5B). The field has increasingly focused on precision medicine strategies that explore advanced genetic screening techniques, molecular targeted therapies, and personalized risk management protocols. Notably, “cervical cancer” and “ovarian cancer” have appeared as significant keywords in the latest period, underscoring the growing recognition of PJS-associated gynecological malignancy as an important research topic. The most recent comprehensive review by Amru and Dhok synthesizes these developments, highlighting the dynamic nature of PJS research and the ongoing efforts to translate molecular insights into clinical practice (Amru and Peutz-Jeghers, 2024). Overall, the bibliometric analysis confirms a dynamic research field evolving from descriptive genetics toward translational studies on surveillance and targeted therapies for PJS-related cancers. This progression represents a critical shift from purely descriptive research to a more integrated, patient-centered approach in understanding and managing this complex syndrome.

#### 3.1.5 Clinical and pathological features of PJS-associated G-EAC

We analyzed 10 female PJS patients with cervical glandular lesions to characterize their clinical profile. Key demographic and clinical manifestations are summarized below (Table 1). Genetic testing confirmed STK11 mutations in two patients, while the remaining eight were diagnosed based on clinical criteria (Laderian et al., 2020). The median age at PJS diagnosis for the cohort was 6 years (range 1–30 years), whereas the median age at diagnosis for aLEGH or G-EAC was 26 years (range 17–51 years).

**Table 1:** Clinicopathological Characteristics of Patients with Peutz-Jeghers Syndrome and Gastric-Type Cervical Adenocarcinoma (n = 10).

Clinicopathological		Mean	Median
characteristics		(SD)	(range)
Age at diagnosis	PJS	9.1 (9.9)	6 (1–30)
(years)	aLEGH/G-EAC	29.5(10.6)	26 (17–51)
		Number	%
Symptoms	Dysmenorrhea/Abdominal		
	/pelvic pain	4	40
	Vaginal bleeding	1	10
	Watery vaginal discharge	3	30
High-risk HPV	Negative	10	100
TCT	Atypical lobulated cervical gland hyperplasia	9	90
	Cervical lesion/hypertrophy		
Ultrasonic	Cystic mass/Pelvic effusion	10	100
Pelvic MRI/CT	G-EAC	6	60
	aLEGH		
Final Pathology	G-EAC and aLEGH	4	40
	IB2	4	40
	IB3	2	20
G-EAC FIGO stage		5	50
		1	10

PJS, Peutz-Jeghers syndrome; G-EAC, Gastric-type Endocervical Adenocarcinoma.

aLEGH, atypical lobular endocervical glandular hyperplasia.

Unlike conventional cervical cancers, our cohort of PJS-associated G-EAC cases exhibited distinct characteristics when compared to typical cervical adenocarcinoma. Notably, all patients were HPV-negative (100 % of cases), underscoring the unique molecular pathogenesis of this disease. Key clinical features included an earlier median age at onset as well as more aggressive behavior relative to typical cervical adenocarcinoma. The most common symptoms associated with G-EAC were vaginal watery discharge, followed by vaginal bleeding, dysmenorrhea, and abdominal or pelvic pain, which were closely correlated with disease progression (Bhardwaj and Kalir, 2023). Diagnostic evaluation revealed distinctive features within our cohort. Initial screening involved comprehensive gynecological examinations combined with HPV testing and ThinPrep cytologic test (TCT) for all cases, revealing no evidence of high-risk HPV infection detected in any patient. TCT results from eight patients revealed atypical lobular hyperplasia of cervical glands. Ultrasound examinations identified cervical space-occupying lesions in all patients (Yang et al., 2024, Yang et al., 2025). Pelvic MRI or computed tomography (CT) scans were performed in eight patients, detecting cystic-solid masses or lesions in five patients and pelvic effusion in six. Pathological confirmation required immunohistochemical profiling to exclude metastatic adenocarcinoma.

Given the rarity and aggressive nature of PJS-associated G-EAC, our management approach diverges from standard protocols for cervical cancer. Surgical resection typically entails a radical hysterectomy accompanied by lymphadenectomy. Adjuvant therapy is considered even for stage I diseases, with adjuvant radiotherapy recommended for G-EAC patients due to their high recurrence rates. Systemic therapy employs platinum-based regimens for advanced disease, with the STK11 mutation status guiding the selection of targeted therapies. Final diagnoses included four cases of aLEGH (with possible gastric-type adenocarcinoma not excluded), four confirmed cases of G-EAC, and two cases diagnosed with both aLEGH and G-EAC. Among the six patients confirmed to have G-EAC, staging revealed advanced early-stage disease, with five cases classified as FIGO stage IB2 and one as IB3.

Close lifelong follow-up is essential for PJS patients because of their increased risk of various cancers and the potential recurrence of G-EAC. The follow-up regimen includes gynecological examination, cervical/vaginal stump cytology every 3–6 months, tumor marker assessments, as well as pelvic and abdominal ultrasound evaluations. Although PET imaging was not routinely employed within our current cohort, we acknowledge its potential clinical utility in enhancing disease characterization for PJS-associated G-EAC. Specifically, PET-CT may provide significant advantages in detecting occult metastatic disease, quantifying metabolic tumor activity, and improving staging accuracy. As part of an enhanced imaging protocol to be implemented in 2025, all study participants will undergo standardized PET-CT evaluation as a fundamental component of their diagnostic workup. A prospective study will be conducted to evaluate the utility of PET imaging in PJS-associated G-EAC and to perform comparative analysis with current imaging modalities.

## 4 Discussion

Peutz-Jeghers syndrome is a prototypical hereditary cancer syndrome, primarily known for its association with gastrointestinal malignancies (Latchford and Clark, 2022, Amru and Dhok, 2024). Our bibliometric analysis reveals a growing interest in the broader cancer spectrum of PJS, particularly gynecological cancers. The increasing number of publications and evolving research hotspots highlight the clinical and scientific significance of PJS-related G-EAC, a rare but aggressive tumor. Keywords such as “hereditary gynecological cancers,” “endometrioid adenocarcinoma,” and “Lynch syndrome” appeared in clusters, reflecting an expanding recognition of PJS’s cancer risk beyond the gastrointestinal tract and comparisons with other familial cancer syndromes. Geographically, research activity is concentrated in North America and Europe, with relatively limited contributions from low- and middle-income countries, potentially reflecting disparities in healthcare infrastructure or patient registry systems.

Several critical research gaps remain. First, the small sample sizes in most studies limit the generalizability of findings and hinder the development of robust clinical guidelines. Second, the molecular mechanisms by which STK11 mutations contribute to the pathogenesis of G-EAC are not yet fully elucidated. Third, ethnic and geographic disparities in disease presentation and treatment outcomes remain understudied, impeding global clinical translation (Jiang et al., 2019).

The management of PJS-associated G-EAC necessitates a tailored diagnostic and therapeutic approach due to its aggressive biology, early metastatic potential, and poor prognosis (Yamamoto et al., 2023). Current management strategies involve transvaginal ultrasound alongside pelvic MRI—utilizing a novel grading system for risk stratification—for detection and staging purposes; this is supplemented by CT imaging to evaluate nodal involvement and distant metastasis. The future integration of PET/CT may further enhance the identification of occult metastasis. Treatment emphasizes radical surgery (hysterectomy ± lymphadenectomy) for localized disease, accompanied by adjuvant chemoradiation in high-risk cases. However, targeted therapies (e.g., mTOR inhibitors) and immunotherapy are currently under investigation due to the STK11/TP53-driven molecular profile characteristic of G-EAC (Yang et al., 2024). Challenges faced include diagnostic delays, limited therapeutic options, and quality-of-life concerns in young patients.

In our case series, imaging played a pivotal role in diagnosis. Transvaginal ultrasound detected cervical masses in all patients, and MRI/CT provided detailed information regarding tumor extent and pelvic fluid. These findings align with the imaging known imaging features of G-EAC, which often presents as large, cystic cervical masses with local infiltration. While PET-CT was not routinely used in our current cohort, we recognize its clinical value for detecting occult metastases, quantifying tumor metabolic activity, and improving staging accuracy (Dzaye et al., 2023). As part of an enhanced imaging protocol that has been implemented, all study participants will undergo standardized PET-CT evaluations as a fundamental component of their diagnostic workup. A prospective study is desighed to assess the utility of this approach in PJS-associated G-EAC compared to current imaging modalities. The coexistence of aLEGH and invasive G-EAC in several cases supports a progression model in which PJS-related cervical neoplasia may originate from these glandular hyperplastic lesions. All G-EAC cases in our series were classified as advanced early-stage (IB2/IB3), consistent with the aggressive clinical behavior of PJS-associated G-EAC. These tumors tend to be less responsive to standard chemoradiation and are associated with higher recurrence rates and reduced survival compared to conventional cervical adenocarcinomas (Nishio et al., 2024, Wang et al., 2023). This underscores the importance of early detection and targeted surveillance in PJS patients, particularly women of reproductive age. Given the resistance of G-EAC to conventional therapies, there is an urgent need to develop novel treatment strategies, including targeted and immunotherapies, based on the molecular profiles of these tumors.

Future directions will emphasize the establishment of multicenter registries, the implementing of advanced screening methods (e.g., MRI surveillance and biomarker studies), and the conduction of clinical trials aimed at optimizing early detection and precision therapy. A multidisciplinary approach is essential, integrating gynecologic, gastrointestinal, and genetic surveillance for malignancies associated with PJS. Our future research will prioritize expanding cohort sizes and integrating advanced technologies such as single-cell RNA sequencing and microbiome profiling to better understand tumor heterogeneity and dysregulated microbial signatures in PJS-related G-EAC. Incorporating multilingual literature may also enhance understanding of this condition across diverse populations.

However, our study has limitations. The retrospective design and small sample size affect statistical power and generalizability. Additionally, the lack of long-term follow-up and detailed molecular profiling in some cases limits mechanistic insights. Nonetheless, our findings contribute to the growing body of evidence supporting the unique clinical and pathological characteristics of PJS-associated G-EAC and emphasize the need for tailored screening and management protocol in this high-risk population.

## 5 Conclusion

In conclusion, this study provides a comprehensive review of the existing literature on Peutz-Jeghers syndrome (PJS) and its association with gynecological malignancies, with a particular focus on gastric-type endocervical adenocarcinoma (G-EAC). This study addresses the critical gap in the understanding of gynecological risks among PJS patients, with a particular focus on non-HPV-driven G-EAC, and provides a basis for the development of individualized clinical strategies. These findings highlight the need for enhanced screening protocols and multidisciplinary management approaches to improve early detection and clinical outcomes. Future research should focus on elucidating the molecular mechanisms underlying STK11 inactivation. Additionally, advanced techniques such as single-cell RNA sequencing and microbiome profiling should be employed to identify the cellular origins and potential therapeutic targets of PJS-associated gynecological malignancies. To ensure global applicability, cross-ethnic and international studies are urgently needed to explore variations in disease patterns and genetic modifiers.

## Author contributions

Niu TH and Cai GQ designed the study, analyzed the data, and wrote the manuscript; Zhang L and Wang HF collected the data; Jia JH was responsible for data analysis and figure creation. All authors have reviewed and approved the final manuscript, confirming their contributions to the article and endorsing the submitted version.

## Data availability statement

The original contributions of this study are presented in both the article and supplementary materials. For additional questions, please contact the corresponding author.

## CRediT authorship contribution statement

**Tianhui Niu:** Writing – original draft, Project administration, Conceptualization. **Jinghui Jia:** Funding acquisition, Formal analysis, Data curation. **Lei Zhang:** Methodology. **Hongfang Wang:** Methodology, Data curation. **Guoqing Cai:** Writing – review & editing, Conceptualization.

## Funding

This work was supported by the funding of General Project of the Special Scientific Research on Military Family Planning (No. 23JSZ06).

## Declaration of competing interest

The authors declare that they have no known competing financial interests or personal relationships that could have appeared to influence the work reported in this paper.
