# Supplementary material for: Peutz-Jeghers syndrome in gynecological cancers: bibliometric trends, clinical insights, and future directions
Source: Gynecol Oncol Rep. 2025 Sep 4;61:101941. doi: 10.1016/j.gore.2025.101941 (PMC12451278; doi:10.1016/j.gore.2025.101941)
Supplement: Supplementary Data 1 [file mmc1.docx]

**Table S1** Top 10 countries and institutions contributing to research on Peutz-Jeghers syndrome and gynecological cancers.

| **Rank** | **Country** | **Counts** | **Institution** | **Counts** |
| --- | --- | --- | --- | --- |
| 1  2  3  4  5  6  7  8  9  10 | USA  JAPAN  ENGLAND  ITALY  PEOPLES R CHINA  AUSTRALIA  CANADA  BRAZIL  NETHERLAND  PORTUGAL | 43 (44.8%)  13 (13.5%)  12 (12.5%)  8 (8.3%)  7 (7.3%)  5 (5.2%)  4 (4.2%)  3 (3.1%)  3 (3.1%)  3 (3.1%) | Univ Warwick  Dana Farber Canc Inst  Univ Alabama Birmingham  Okayama Univ  Boston Univ  Jilin Univ  Ut Md Anderson Canc Ctr  Univ Brimingham  Harvard Univ  Massachusetts Gen Hosp | 9 (9.4%)  5 (5.2%)  4 (4.2%)  4 (4.2%)  3 (3.1%)  3 (3.1%)  3 (3.1%)  3 (3.1%)  3 (3.1%)  3 (3.1%) |

**Table S2** Top 10 co-cited references in research on Peutz-Jeghers syndrome and gynecological cancers.

| **Rank** | **Co-cited Reference** | **Citations** |
| --- | --- | --- |
| 1  2  3  4  5  6  7  8  9  10 | Giardiello Fm, 2000, Gastroenterology, V119, P1447[14]  Hemminki A, 1998, Nature, V391, P184[15]  Tan Mh, 2012, Clin Cancer Res, V18, P400[16]  Beggs Ad, 2010, Gut, V59, P975[17]  Van Lier Mgf, 2010, Am J Gastroenterol, V105, P1258[18]  Hearle N, 2006, Clin Cancer Res, V12, P3209[19]  Jenne De, 1998, Nat Genet, V18, P38[20]  Antoniou A, 2003, Am J Hum Genet, V72, P1117[21]  Syngal S, 2015, Am J Gastroenterol, V110, P223[22]  Klein Ap, 2004, Cancer Res, V64, P2634[23] | 29  22  20  18  17  16  14  13  13  13 |

**Table S3** Top 10 journals and co-cited journals in research on Peutz-Jeghers syndrome and gynecological cancers.

| **Rank** | **Journal** | **Counts** | **IF** | **Co-cited Journal** | **Co-citation** | **IF** |
| --- | --- | --- | --- | --- | --- | --- |
| 1  2  3  4  5  6  7  8  9  10 | International Journal of Molecular Sciences  Cancer Journal  Obstetrics and Gynecology Clinics of North America  Radiographics  Dalton Transactions  Plos One  World Journal of Gastroenterology  Histopathology  Journal of Obstetrics and Gynaecology Research  Pancreatology | 3 (3.1%)  3 (3.1%)  3 (3.1%)  3 (3.1%)  3 (3.1%)  3 (3.1%)  2 (2.1%)  2 (2.1%)  2 (2.1%)  2 (2.1%) | 4.9  2.6  2.6  5.27  3.5  2.9  1.09  4.2  1.7  2.8 | J Clin Oncol  Cancer Res  Gynecol Oncol  Nature  New Engl J Med  Gastroenterology  J Med Genet  Clin Cancer Res  Nat Genet  Gut | 311  227  188  170  169  168  157  152  148  139 | 42.1  12.5  4.5  50.5  96.2  25.4  3.5  10  31.7  23 |

**Table S4** Top 20 keywords in research on Peutz-Jeghers syndrome and gynecological cancers.

| **Rank** | **Keywords** | **Counts** | **Rank** | **Keywords** | **Counts** |
| --- | --- | --- | --- | --- | --- |
| 1  2  3  4  5  6  7  8  9  10 | peutz-jeghers syndrome  lynch syndrome  lkb1  surveillance  familial pancreatic cancer  ovarian cancer  brca  screening  hereditary cancer syndrome  cervical cancer | 11  10  8  6  6  6  5  5  5  4 | 11  12  13  14  15  16  17  18  19  20 | genetic testing  family history  hereditary breast and ovarian cancer syndrome  pancreatic cancer  hereditary breast cancer  risk reduction  high risk  germline mutation  stk11  tp53 | 4  4  4  4  4  3  3  3  3  3 |
